# A Nationwide Social Contact Survey Dataset for Public Health and Social Sciences Research in South Korea

**DOI:** 10.1038/s41597-026-06896-y

**Published:** 2026-03-05

**Authors:** Min-Kyung Chae, Woo-Sik Son, Kyeongah Nah, Minsoo Kim, Jong-Hoon Kim, Jonggul Lee

**Affiliations:** 1 Research team for transmission dynamics of infectious diseases, National Institute for Mathematical Sciences, 70, Yuseong-daero 1689 beon-gil, Yuseong-gu, 34047 Daejeon Republic of Korea; 2 Busan Center for Medical Mathematics, National Institute for Mathematical Sciences, 179, Gudeok-ro, Seo-gu, 49241 Busan Republic of Korea; 3 Department of Electronics Communications Engineering, National Korea Maritime & Ocean University, 727 Taejong-ro, Yeongdo-gu, Busan 49112 Republic of Korea; 4 Epidemiology, Public Health, Impact, International Vaccine Institute, 1 Gwanak-ro, Gwanak-gu, Seoul 08826 Republic of Korea

**Keywords:** Public health, Sociology

## Abstract

Social contact data underpin research across public health, social and behavioral sciences, and network analysis, as interpersonal interactions shape population dynamics and societal outcomes. In South Korea, previous social contact surveys have been limited by small samples and restricted accessibility, often necessitating reliance on synthetic data from international studies. To address this gap, we conducted a large-scale national contact survey during winter 2023–24. A total of 2,415 individuals were recruited across age groups and regions, resulting in a final sample of 1,987 participants. The survey captured daily close contact behaviors during weekdays, weekends, school vacations, and holidays, along with demographic and contextual details. This study emphasizes transparency by documenting the entire process—from survey design to rigorous data cleaning and validation. The dataset provides comprehensive evidence on post-pandemic contact patterns in South Korea and supports applications in infectious disease modeling, public health policy analysis, and social network research. By sharing the methodology and dataset, we aim to establish a reproducible framework for future social contact surveys in South Korea and beyond.

## Background & Summary

Understanding social contact patterns is fundamental across multiple disciplines, including social sciences, behavioral science, and public health. These patterns reveal how interpersonal relationships and social structures influence behaviors, health outcomes, social inequalities, cultural practices^1,2^. In recent decades, social contact studies have become especially important in infectious disease modeling, providing the empirical basis for estimating transmission parameters and evaluating intervention strategies^3–9^.

The COVID-19 pandemic highlighted the need for accurate, context-specific social contact data, as changes in daily interactions and mobility patterns had profound implications for disease dynamics and public policy^10,11^. In South Korea, however, previous contact surveys have been constrained by small sample sizes^12,13^ and limited public accessibility^14^, leading researchers to rely heavily on synthetic contact data^5^ derived from international studies—most notably POLYMOD^8^—for mathematical modeling^15–19^. These limitations have reduced the accuracy and applicability of epidemiological models tailored to Korean demographic and socio-economic contexts.

To address these gaps, we conducted a national-level representative social contact survey in South Korea, recruiting 2,415 participants stratified across age groups and geographic regions during two separate periods (December 2023 and February 2024). The study collected detailed demographic information and comprehensive data on each reported contact, including household composition, contact characteristics, relationship type, meeting location, and interaction patterns. Participants recorded their contacts using either online or paper-based diaries, ensuring accessibility across different age groups and levels of digital literacy. All data underwent rigorous quality control procedures, including duplicate removal, logical consistency checks, typographical error corrections, and reclassification of ambiguous responses. All procedures were designed to maximize transparency and reproducibility.

A previous analysis of this dataset revealed distinctive features of social mixing in South Korea^20^. Notably contacts with extended family members increased substantially during holiday periods. Moreover, the age-specific contact patterns demonstrated strong assortative mixing, with notably higher contact rates among elderly populations compared to other countries. These findings underscore the value of the dataset for infectious disease modeling and public health policy development. Specifically, the data enables construction of age-specific contact matrices, which are essential for estimating basic reproduction numbers (*R*_0_)^21^. The detailed context-specific contact data facilitate the design of targeted intervention strategies that account for variations in interaction patterns by age group or location^22^. Public health authorities can leverage these insights to evaluate the effects of hygiene promotion efforts, identify high-contact populations requiring prioritized interventions, and analyze differences in contact behaviors driven by socio-economic factors^23,24^.

The survey design, data processing procedures, and anonymized dataset are openly shared to support reuse in infectious disease modeling, social science research, and to provide a methodological reference for future contact survey studies in South Korea and internationally.

## Methods

### Survey design

The study was designed to measure the frequency and patterns of close social contacts among individuals in South Korea in the post-pandemic era. The definition of close social contact and the survey structure were adapted from Mossong *et al*.^8^ and modified to fit the Korean context. All survey procedures, including participant recruitment and data collection, were conducted by Gallup Korea, a professional survey research organization.

In the study, close social contact was defined as either physical contact involving skin-to-skin interaction or non-physical contact characterized by a conversational exchange of at least three words without skin contact. Participants recorded the individuals they had close social contact with each day for a single day. If they had contact with the same person multiple times in one day, they were instructed to consolidate all instances into a single entry. The survey was conducted in two separate rounds, each lasting one week (Round 1: 2023/12/06 to 2023/12/12; Round 2: 2024/02/07 to 2024/02/13). The second survey period included a major traditional holiday in South Korea (February 9–12, 2024).

Participants responded to two kinds of surveys (Table 1). First, they completed a preliminary survey collecting basic sociodemographic information prior to participating in the main survey. This survey consisted of six questions in total, combining short-answer and multiple-choice formats. Among them, two questions about age (SQ1, DQ2-1~10) and one question about the number of cohabiting family members (DQ2) were short-answer questions, where participants could enter only numerical values. The remaining four questions were multiple-choice. After completing the preliminary survey, participants then took the main survey, which they were required to complete daily for 14 days (7 days per round, twice). This survey consisted of nine questions using three response formats: short-answer questions, multiple-choice questions, and multiple-choice questions with a free-text option. The short-answer questions included age (Q1) and contact group size (Q9), where participants could only enter numerical values. The multiple-choice questions with a free-text option covered relationship with the contact person (Q4) and contact location (Q5). Participants were required to choose one option from the provided list, but if none of the predefined choices applied, they could enter their own response. Similarly, for contact locations, they selected all relevant places where they had social interactions throughout the day, with the option to provide additional details if necessary. The remaining questions followed a standard multiple-choice format, allowing participants to select the most appropriate response from the given options.

**Table 1:** The question lists of the preliminary survey and main survey. There are each 6 and 9 questions. The preliminary survey is questions about sociodemographic of participants. The main survey is questions about social close contact behaviors.

	Question	Answer
SQ1	What year were you born?	Answer Format 1
SQ2	What is your sex?	Answer Format 2
SQ3	Which region do you currently reside in?	Answer Format 2
DQ1	What is your occupation?	Answer Format 2
DQ2	How many people in your household are currently living with you? (Including yourself)	Answer Format 1
DQ2-1~10	Please record the ages of all cohabiting members. (record up to a maximum of 10)	Answer Format 1
Q1	What is the age of the contact person? If you don’t know exactly, please record an estimate.	Answer Format 1
Q2	What is the sex of the contact person?	Answer Format 2
Q3	Did you have a conversational exchange of three or more words, or did you have a skin contact, or both?	Answer Format 2
Q4	Which of the following best describes your social relationship with the contact person?	Answer Format 3
Q5	Where did you meet the contact person? Please select all the locations if you had multiple contacts with one person on the same day.	Answer Format 3
Q6	Did the contact person live in the same residence region as you?	Answer Format 2
Q6-1	Where did the contact person live?	Answer Format 2
Q7	How often do you meet with the contact person?	Answer Format 2
Q8	How much of the day did you spend with the contact person? Please answer the total duration if you had multiple contacts with one person on the same day.	Answer Format 2
Q9	What was the size of contact with the contact person? The size of contact means the total number of contact people, including yourself. Please answer the total size of contact if you had multiple contacts with one person on the same day.	Answer Format 1

^*^Answer Format 1: short-answer question, Answer Format 2: multiple-choice, Answer Format 3: multiple-choice question with a free text option.

The survey employed two methods for data collection to accommodate participant preferences and accessibility: online and paper diaries. Participants using the online diary accessed the provided web address to record their sociodemographic information and daily contact data. This approach offered convenience by allowing participants to complete the survey from any location using a laptop or mobile phone. However, once recorded, information was difficult to edit online diary. A paper-based diary option was therefore provided for participants with limited access to or familiarity with internet-based platforms, mostly young children or older adults. While the paper diary was easy to edit and did not require an internet connection, participants had to be careful not to misplace the diary. Each participant was provided with a single paper diary. Survey staff delivered the paper diaries in person at the beginning of the survey and collected them directly upon completion. The data collection methodology was stratified by age groups to ensure an appropriate response mechanism. Participants aged 19–64 years used the online diary (68.65%). For minors aged 0–14, the paper diary was employed, with their parents or guardians filling it out on their behalf (12.08%). Adolescents aged 15–18 and seniors aged 65–79 completed the paper diary themselves (3.82% and 15.45%, respectively).

### Participants

The recruitment period spanned from November 15 to 30, 2023, in South Korea. Recruitment was conducted exclusively online and followed a quota sampling design stratified by geographic region (five provinces) and age (10-year intervals), with sex not included as a stratification variable. The mid-year population distributions by age and region for South Korea in 2023 are provided in the Supplementary Material.

All participants received a comprehensive explanation of the study protocol, including their right to withdraw at any time without penalty. For children aged 6 years or younger, written informed consent was obtained from a parent or legal guardian. For minors aged 7–18 years, both parental/guardian written consent and the child’s assent were required. In addition, for participants aged 7–12 years, a separate assent form written in age-appropriate language was provided to ensure comprehension. Adults provided their own written informed consent prior to enrollment.

The consent form specified that personal identifiers (e.g., name and contact details) would be removed after achieving the purpose of collection and were permanently deleted, and that the remaining survey data would be stored and analyzed only in anonymized form under appropriate safeguards. It further explained that the anonymized research data would be used for this study and for reporting its scientific results, and that personally identifiable information would not be disclosed to third parties. The study protocol, including the collection and use of anonymized data for research, was approved by the Public Institutional Review Board (P01-202310-01-056).

For participants aged 0–14 years, parents or guardians completed the diary entries on behalf of their children. Parents or guardians were instructed to ask their children directly about all contacts they had during the day and to record the reported interactions using age-appropriate guidance. Each proxy respondent was allowed to complete the diary for only one child. The survey did not include a separate section to record demographic information (e.g., age or sex) of proxy respondents.

A total of 2,415 participants completed the consent form and the preliminary demographic survey. Of these, 428 participants were excluded, resulting in the final dataset of 1,987 participants (52.1% female, average age = 42.01 ± 19.77 years, age range = 0–79, see Supplementary Material). Foreigners were not included in the study.

To encourage full participation, a small monetary incentive was provided to participants who completed the preliminary survey and both rounds of the main survey. The incentive was distributed after all survey activities had been concluded.

## Data Records

The dataset is available at Figshare^25^. It consists of two anonymized comma-separated value (CSV) files and one questionnaire file. All research data have been anonymized prior to release.

The files ‘code_book_preliminary_survey.xlsx’ and ‘code_book_main_survey.xlsx’ provide codebooks for the preliminary and main survey datasets, respectively. For each variable in the corresponding CSV file, these codebooks list the column name, the original survey question text, the response options and coding scheme (including numeric codes used for categorical variables), and any recoding or standardization applied during data cleaning. They therefore serve as the primary reference for interpreting the column headings and response categories in the data files.

The file ‘results_preliminary_survey.csv’ contains the line-list data from the preliminary survey, with one row per participant. Columns include a unique participant identifier and sociodemographic variables such as age, sex, region of residence, occupation, household size, and the ages of cohabiting family members, corresponding to the preliminary survey questions. The file ‘results_main_survey.csv’ contains the line-list data from the main contact diary survey, with one row per reported close contact. Its columns include the participant identifier linking to the preliminary survey, the contact person’s age and sex, relationship category, contact location (with multiple locations recorded where applicable), frequency and duration of contact, and contact group size, matching the main survey items. The file ‘survey_offline_v3_ENG.pdf’ contains the English version of the questionnaire used for offline data collection and shows the exact wording and ordering of the survey items.

## Data Overview

In total, 1,987 participants contributed 133,776 recorded close social contacts over two survey weeks. The first survey round contained 69,150 contacts and the second round 64,626 contacts, corresponding to average numbers of 34.8 (standard deviation 24.55) and 32.5 (standard deviation 20.15) contacts per participant per week, respectively. The maximum number of contacts reported by a single participant over both weeks was 329, and the minimum was 2.

## Technical Validation

**Fig. 1:**
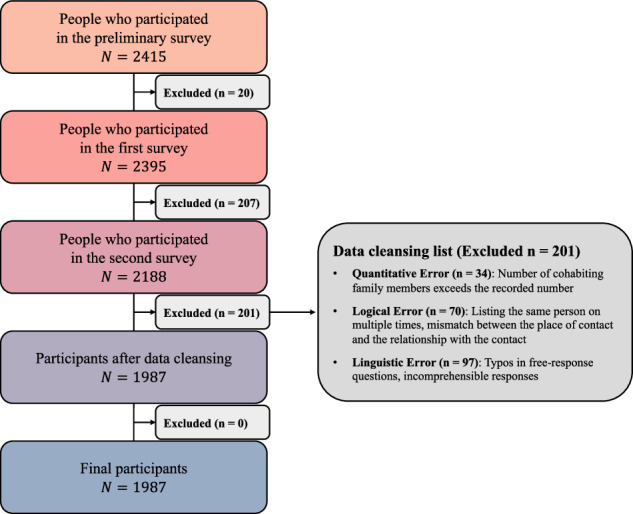
Schematic of the procedure for extracting the final list of survey participants. The datasets underwent extensive cleansing processes to ensure accuracy and reliability.

Figure 1 illustrates the flow of participants through the study, from initial recruitment and survey completion to data cleaning and the final analytic dataset. A total of 2,415 individuals expressed willingness to participate and completed the preliminary survey. Of these, 20 participants were excluded, and 2,395 participants completed the first round of the main survey. Among first-round completers, 207 participants were excluded, resulting in 2,188 participants who completed the second round of the main survey. Data cleaning procedures were applied to 2,188 participants who completed the preliminary survey as well as both rounds of the main survey. After data cleaning, the final analytic survey dataset comprised 1,987 participants.

The datasets underwent extensive cleaning processes to ensure accuracy and reliability. To ensure the reliability and integrity of our contact survey dataset, we implemented a comprehensive data validation and cleaning protocol. This protocol involved systematic identification and correction of errors across multiple dimensions, including quantitative inconsistencies, logical errors, and linguistic inaccuracies. All validation procedures were carefully documented and only applied when errors could be unambiguously identified, thus preserving the integrity of the original data where uncertainty existed.

### Quantitative errors

We identified the cases where participants recorded daily contacts with cohabiting family members that exceeded the number of family members reported in the preliminary survey, then excluded participants who did not fit the description of cohabiting family members. After reviewing these discrepancies, we excluded participants whose contact patterns did not align with their reported household composition, as these inconsistencies suggested potential misunderstanding of survey instructions or erroneous data entry.

### Logical errors

**Fig. 2:**
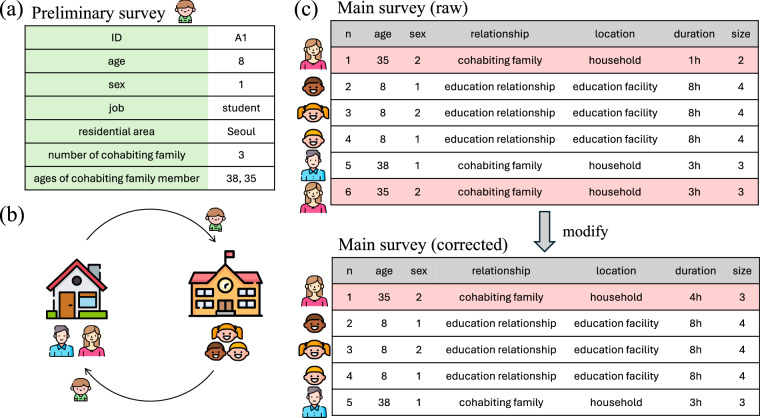
Diagrams of the procedure for logical errors. (**a**) Results of the participant of the preliminary survey. (**b**) Illustration of a participant’s contacts for a day. (**c**) Schematic of how modify for duplicate. The presumed mother (shaded in red) is entered in two lines, so the two is merged into one.

Our survey instructed participants to record each unique contact person in a single row, regardless of how many times they contacted the same person throughout the day. We corrected the cases where the same person was recorded on multiple rows. For example, if a participant recorded meeting their mother in the morning and then again after returning from school, these cases were merged into a single record (Fig. 2). Only clear duplicates, cases where the same person can be unambiguously identified, were addressed since names of contact people were not collected. We identified clear duplicates by cross-referencing the age and sex of the participants’ cohabiting family member from the preliminary survey and the main survey. When the participants entered multiple entries for the same cohabiting family member, we consolidated into a single entry.

We also standardized relationship. In some cases, participants selected the “etc” category for relationships, even when more specific options like a “friend” category were available. In this survey, “friend” was intended to include acquaintances or regular contacts. However, due to cultural nuances in South Korea, many participants seemed to interpret “friend” narrowly to include only close relationships maintained over a long period of time. As a result, we reclassified responses such as “neighbor” or “partner” recorded under “etc” as “friend”. Additionally, the relationships were further subdivided for clarity (see the codebook in Supplementary Material).

Similarly, location and occupation data were standardized. For example, for a 9-year-old participant who is an elementary school student, an education-related contact occurring at “workplace”, we corrected the contact place to “educational facility”. Regarding occupations, we reclassified specific occupations recorded under “etc” to the appropriate category in the multi-choice question. For example, “lecturer” was reclassified as “professional/freelance worker”.

In the revised survey results, we now clarify that we did not delete any free-text responses submitted under the “etc.” category. All raw free-text (Korean) entries are preserved and released in the public dataset. Because participants could enter a free-text response only after explicitly selecting the “etc.” option, the original responses remain clearly identifiable. For Q4 (social relationship) or Q5 (location), which are single-response items, cases in which the variable “Q4_etc” contains a text entry while the main Q4 variable includes one of the predefined categories (no. 1–6) can be readily recognized as researcher-applied recoding. This structure ensures that users can easily distinguish between original responses and harmonized categories. In addition, as suggested, we now provide both the raw relationship variable and the recoded variable in the dataset, along with a mapping table included in the Supplementary Material.

### Linguistic errors

Responses to free-text fields in multiple-choice questions occasionally contained typographical errors or incomprehensible answers. We corrected obvious typographical errors while preserving the intended meaning. In cases where responses were severely incomprehensible, we excluded these participants from the dataset.

In total, 201 participants were excluded during the validation process: 34 due to quantitative errors, 70 due to logical errors, and 97 due to linguistic errors. Participants with more than one type of error were assigned to the linguistic error category, and therefore no participant was counted more than once. The age and regional distributions of the excluded participants were broadly consistent with the corresponding national distributions in South Korea, indicating that exclusions were not biased. Detailed distributions are provided in the Supplementary Material.

It is important to note that all cleaning processes were performed only when errors could be clearly identified. Furthermore, the age system in South Korea increases an individual’s age by one year on January 1 of each year, regardless of the individuals’ birthdate. While we explicitly requested participants to report their age in full years (international age), there might be a one-year age gap between the two survey periods (2023 and 2024), particularly for cohabiting family members. We accounted for this potential variation during our analysis.

## Usage Notes

This dataset is provided in CSV format and can be easily accessed and analyzed using standard statistical software such as R or Python. Researchers interested in constructing age-stratified contact matrices may find the R package ‘socialmixr’ helpful, and the dataset structure is compatible with common epidemiological modeling workflows. We recommend applying appropriate demographic weights based on the 2023 Korean mid-year population for population-level analysis. All data cleaning and error correction procedures have already been completed, as described in the Technical Validation section. Therefore, the dataset is ready for direct use in most research applications.

For further data processing—such as aggregating contacts by age group, relationship type, or location, or constructing custom contact matrices—users should consult the accompanying codebook. The codebook provides detailed definitions for each variable, clarifies response categories, and explains how multiple-choice and free-text responses were coded. In particular, some survey questions included an “etc” (other, please specify) option, allowing participants to provide free-text responses if none of the predefined choices applied. These free-text entries are written in Korean only, and no English translation is provided. Because participants often entered highly variable and subjective answers in these fields, systematic translation or reclassification is challenging, and the majority of analyses should focus on the predefined response categories, which are fully documented in English. If your research requires the use of the “etc” fields, you may need assistance from a Korean speaker or consider using automated translation tools; however, interpretation may be limited, and consistency cannot be guaranteed. In many cases, excluding the “etc” entries from quantitative analysis may be the most practical approach.

Beyond its primary application in infectious disease modeling, this dataset offers significant value for social sciences and interdisciplinary research. Because it includes not only detailed demographic variables (such as age, sex, residence, and occupation) but also contextual information on the day type of the contact (weekdays, weekends, school vacations, and holidays), relationship type, and contact location, it can be used to address a wide range of questions in network science, behavioral research, and urban studies. For example, it enables social network analysis to visualize and quantify the structure of interpersonal relationships within the population^26^. When combined with other data sources such as large-scale communication data^27,28^,emotionally close relationships within individual social networks over time and space.

Despite these strengths, several limitations should be considered when using this survey dataset. First, participants may have experienced reporting fatigue, and some may not have consistently recorded their daily contacts. We cannot rule out the possibility that recall bias and reporting fatigue led to a gradual decline in the number of reported contacts over time (see the Supplementary Material). However, because the survey covered distinct calendar periods, this decline may also reflect genuine temporal variation or changes in participant adherence. In addition, the survey did not structure daily recall by time-of-day segments (e.g., morning, afternoon, evening), which may have increased the likelihood of omitted contacts compared to chronologically segmented diary designs. Notably, recent methodological work by Dan, S. *et al*.^29^ has proposed approaches to correct for such biases in contact diary data. Future studies will explore the application of these methods to adjust contact estimates derived from this dataset. Second, the survey employed a one-person–one-row structure in which participants recorded each contacted person only once per day, consolidating multiple encounters. As a result, within-day variation in contact timing or intensity cannot be recovered from the dataset. Users interested in encounter-level heterogeneity should take this limitation into account. Third, due to the nature of the survey, all responses rely on subjective self-assessment. For this reason, some answers listed under the “etc.” category were inconsistent or difficult to interpret. It should also be noted that no external validation of these responses has been conducted. Fourth, contact data for children were collected via proxy reporting by parents or guardians, which may introduce additional measurement error. Despite explicit instructions for proxy respondents to ask children directly about all contacts, under-reporting or misclassification of contacts—particularly among older children with more independent daily activities—cannot be ruled out. Fifth, because the survey was conducted anonymously, it was not possible to analyze how frequently participants met the same individual over the course of a week. We were only able to measure the total number of reported contacts, without the ability to trace individual contact trajectories. However, participants were asked to indicate how frequently they interacted with each contact, which may provide partial insights. Furthermore, information on response rates, refusal patterns, and the number of contact attempts was not available from the survey vendor and therefore could not be reported, which may limit the assessment of potential selection bias. Finally, although the sample of 1,987 participants is statistically sufficient for many analyses, it may not fully represent the entire Korean population of approximately 55 million.

### Ethics statement

This study was approved by the Public Institutional Review Board (P01-202310-01-056). Written informed consent for participation and for the use of anonymized survey data in scientific research was obtained from all adult participants. For children and adolescents, written informed consent was obtained from a parent or legal guardian, in addition to age-appropriate assent from the child where applicable. All personal identifiers were permanently deleted after achieving the purpose of collection, and personally identifiable information was not disclosed to any third parties.

## Supplementary information

Supplementary Material

Supplementary information

Supplementary information

## Data Availability

The dataset is available at Figshare (10.6084/m9.figshare.29312222). It consists of two anonymized CSV files (“results_preliminary_survey.csv” and “results_main_survey.csv”) containing the line-list data from the preliminary and main contact diary surveys, respectively. A PDF file of the offline questionnaire (“survey_offline_v3_ENG.pdf”) and two Excel codebooks (“code_book_preliminary_survey.xlsx” and “code_book_main_survey.xlsx”) are also provided to support data interpretation and reuse.

## Code availability

No custom code was used beyond standard data cleaning and descriptive analyses, which are fully described in the Methods and Technical Validation sections. Accordingly, no standalone software or analysis package has been generated that would require a separate code repository.
