# Supplementary material for: A Nationwide Social Contact Survey Dataset for Public Health and Social Sciences Research in South Korea
Source: Sci Data. 2026 Mar 5;13:603. doi: 10.1038/s41597-026-06896-y (PMC13079890; doi:10.1038/s41597-026-06896-y)
Supplement: Supplementary file 1 — Supplementary Material [file 41597_2026_6896_MOESM1_ESM.pdf]

Supplementary Material

Contents

List of Tables

S1 Mid-year population distribution of South Korea (2023) 2
S2 Demographic characteristics of all consented participants (N = 2,415) 2
S3 Demographic characteristics of the final analytic sample (N = 1,987) 4
S4 Demographic characteristics of excluded participants (N = 201) 4
S5 Mapping of relationship categories from free-text responses 5
S6 Mapping of contact location categories from free-text responses 6
S7 Daily average number of reported contacts per participant 7

Supplementary Material

This Supplementary Material provides additional tables and documentation supporting the survey dataset, sample representativeness, data validation, and variable harmonization procedures described in the main text.

Supplementary Tables

Table S1. Population distribution of South Korea in 2023

	0-9	10-19	20-29	30-39	40-49	50-59	60-69	70-79
Metropolitan	1,748,880	2,089,142	3,689,471	3,695,458	4,154,401	4,268,398	3,517,498	1,737,774
Chungcheong	398,620	481,945	705,196	683,779	855,990	902,258	812,213	414,906
Gangwon	93,462	116,426	180,098	158,575	211,281	259,575	272,412	139,577
Youngnam	815,493	1,010,696	1,493,388	1,425,704	1,894,448	2,169,935	2,052,608	1,087,314
Honam	373,796	488,731	699,731	596,773	830,838	961,881	853,990	501,190

Table S1: Mid-year population distribution of South Korea in 2023 by age group and region. Population counts were obtained from official national statistics and were used as reference distributions for quota sampling and post-stratification weighting.

This table presents the national population distribution by age group and geographic region in South Korea for 2023. These distributions were used to guide quota sampling during recruitment and to construct demographic weights for population-level analyses.

Table S2. Demographic characteristics of all consented participants (N = 2,415)

	Characteristics	No. of participants
Age group	0-9	164
	10-19	210
	20-29	348
	30-39	371
	40-49	422
	50-59	396
	60-69	326
	70-79	178
Sex	Male	1,174
	Female	1,241
Region	Metropolitan	1,248
	Chungcheong	245
	Gangwon	85
	Youngnam	584
	Honam	253
Household size	1	315
	2	534
	3	707
	4	684
	5+	175

Table S2: Demographic characteristics of all individuals who completed the informed consent form and preliminary survey (N = 2,415). Variables include age group, sex, and region of residence.

This table summarizes the demographic characteristics of all participants who provided informed consent and completed the preliminary demographic survey, prior to any exclusions or data cleansing.

Table S3. Demographic characteristics of the final analytic dataset (N = 1,987)

This table describes the demographic composition of participants included in the publicly released dataset. These participants completed the preliminary survey and both rounds of the main contact diary survey and passed all data validation criteria.

Table S4. Demographic distribution of excluded participants (N = 201)

This table presents the age and regional distributions of participants excluded due to quantitative, logical, or linguistic inconsistencies. The distributions are broadly comparable to national population patterns, indicating that exclusions were not systematically biased.

Table S5. Mapping of relationship categories from free-text responses

This table documents the recoding rules applied to free-text responses for social relationships. Original raw responses are preserved in the dataset, and this mapping table enables transparent identification of researcher-applied harmonization.

Table S6. Mapping of contact location categories from free-text responses

This table describes how free-text location entries were standardized to improve consistency while preserving the original responses in the released dataset.

Table S7. Average number of contacts per participant

This table presents the daily mean number of reported close contacts per participant across the 14 survey days, along with 90% confidence intervals. The summaries provide descriptive context for temporal variation in reported contact counts over the study period.

A gradual decline in the average number of reported contacts is observed across survey days. However, this pattern should be interpreted with caution. Because the survey spans distinct calendar contexts—including weekdays, weekends, and a major national holiday—it is not possible to disentangle potential reporting fatigue from genuine temporal variation in social contact behavior. Accordingly, these summaries are presented for descriptive purposes only and should not be interpreted as evidence of causal trends.

Supplementary Notes

Note S1. Representativeness and exclusions

Comparisons across Tables S2–S4 indicate that the demographic distributions of the final analytic dataset and the excluded participants closely resemble the national population structure of South Korea in 2023. This supports the conclusion that data exclusions did not introduce substantial demographic bias.

Note S2. Transparency of recoding procedures

All free-text responses recorded under the "etc." option were retained in the released dataset. Recoding was performed only when responses could be unambiguously mapped to predefined categories. Both raw and recoded variables are provided, along with the mapping tables (Tables S5 and S6), to ensure full transparency and reproducibility.

Note S3. Definition of geographic regions

For analysis and sampling purposes, the 17 administrative divisions of South Korea were aggregated into five geographic regions as follows:

	Characteristics	No. of participants
Age group	0-9	144
	10-19	179
	20-29	265
	30-39	292
	40-49	352
	50-59	316
	60-69	273
	70-79	166
Sex	Male	951
	Female	1,036
Region	Metropolitan	1,047
	Chungcheong	190
	Gangwon	66
	Youngnam	476
	Honam	208
Household size	1	1,047
	2	190
	3	66
	4	476
	5+	208

Table S3: Demographic characteristics of the final analytic dataset after all exclusions and data validation procedures (N = 1,987).

	Characteristics	No. of participants
Age group	0-9	17
	10-19	23
	20-29	31
	30-39	33
	40-49	27
	50-59	30
	60-69	30
	70-79	10
Sex	Male	92
	Female	109
Region	Metropolitan	95
	Chungcheong	27
	Gangwon	3
	Youngnam	53
	Honam	23
Household size	1	34
	2	37
	3	57
	4	53
	5+	20

Table S4: Demographic characteristics of participants excluded during the data validation process (N = 201).

Category	Mapping of relationship categories from free-text responses
Cohabiting family	기숙사 룸메, 룸메이트, 기숙사-금일 집 방문
Extended family	형, 누나, 언니, 오빠, 동생, 남동생, 여동생, 자녀, 엄마, 친정엄마, 아빠, 할머니댁, 이모, 삼촌, 처친척, 친형, 친언니, 동생딸, 친척동생, 동생부부, 친척, 비동거 동생, 비동거 자녀, 비동거친족, 형부, 결혼한 언니, 부모님, 딸(별거), 명절집을 방문한 친지, 비동거가족 남편, 출가한 딸, 결혼하여 따로 사는 딸, 비동거가족(어머니), 고향 가족과 처가 식구, 본가 어머니, 독립해서 살고 있는 자녀, 사돈, 분가한 딸, 분가한 사위, 따로 사는 딸, 처남, 제부, 분가한자녀, 분가한 자부, 주말부부남편, 주말부부, 누님, 가족(아버지), 아버지, 큰 딸, 자취하는 딸, 가족(엄마), 비동거가족, 가족(친형), 가족(형), 형제, 형제(따로 삶), 친정 오빠, 가족, 따로 거주중인 어머니, 친정 엄마, 본가 거주 가족, 가족(어머니), 어머니, 분가한 아들, 분가한 자녀, 분가한 손녀, 서울에 사는 큰 딸, 모친, 독립한 아들, 출가한 아들, 아들, 타지역 딸, 별거한 딸, 딸, 손주, 손녀, 조카, 손자, 자녀, 타지역 교육, 따로사는 딸, 세대분리된 가족, 독립해서 살고 있는 자녀
Coworker	직장상사, 직장동료, 직장, 직장 상사, 직장 주임, 회사 이사, 회사 직원, 팀장, 날 고용한 회사 직원, 날 일용직으로 고용한 회사의 직원, 내가 일용직으로 있던 회사와 현장에서 협업하는 회사의 사장, 같은 일용직 동료, 알바 동료, 연구실 소속 동료, 인턴, 직장사장, 직장 사장, 직장 알바생, 직장내 교육생, 같은 연구실 소속 연구원, 아르바이트 사장님, 아르바이트 사모님, 사업파트너, 센터 동료, 알바 인사담당자, 알바 가는 셔틀기사, 알바 인사담당 및 셔틀기사, 점장님, 직장 어르신, 직장어르신
Classmate	학교누나, 학교형, 아이의 학교 선배, 방과후 축구반, 어린이집친구, 유치원친구, 학원친구, 학원팀, 학원언니, 어린이집언니, 어린이집오빠, 형(학원), 학원동생, 학원형, 학원 선배, 학원 형, 복지관수업친구, 센터친구, 피아노학원 친구, (수학)학원친구, 학원 친구, 복지관수업형, 학원후배, 학원동료, 학원 동료, 직업훈련기관 동기, 직업 훈련 기관 동기, 복지관수업동생, 태권도 형, 학원 동생, 학원 누나, 드럼학원동생, 태권도 동생
Classmate (+ age option)	선배, 선배님, 대학교 선배, 학교선배, 학교 선배, 후배, 학원후배, 학교 후배, 대학후배, 학교후배, 대학원 연구실 선배, 대학교 후배, 후배들, 제자
Brethren	신도 자녀, 교회 집사님, 목사님, 교회 목사님, 목사, 신부님, 스님, 전도사님, 교회 사모님, 교회 목사, 교회 선생님, 삼막사 절 스님, 서광사 절 직원, 종교인
Friend	키즈카페친구, 동네친구, 이웃친구, 친구, 고향친구, 이성친구, 여자친구, 남자친구, 남친, 운동 친구, 운동친구, 친구이자 딸 과외선생님, 운동메이트, 이웃 친구, 친한 언니, 같이 운동하는 친한 동생, 친한 동네 언니&딸 친구, 친한동생, 친한 형, 친한 지인, 아는 여직원, 아는사람, 동네아는분, 아는동생, 병원 아는 사람, 아는 언니, 아는 동생, 아는 아저씨, 지인, 동네지인, 거래처 지인, 동네 지인, 병원 지인, 헬스장 지인, 전직장 업계 지인, 동호회지인, 업무 관련 지인, 사회지인, 모임 지인, 업무상 지인, 단순 지인, 옆집지인, 사우나 지인, 농장 지인, 같은 아파트 사는 지인, 전직장동료, 과거직장동료, 옛직장동료, 은사님, 동호회, 동호회원, 동호회 회원, 모임, 친목모임, 운동 모임, 연인, 애인
Education relationship	선생님, 학교선생님, 담임선생님, 교사, 어린이집 선생님, 유치원 선생님, 초등학교 선생님, 중학교 교사, 방과후 선생님, 학원 선생님, 과외선생님, 학습지 선생님, 피아노 선생님, 미술 선생님, 체육 선생님, 교수, 지도교수, 강사, 외부강사, 돌봄선생님, 복지관 선생님
Hospital relationship	의사, 간호사, 간호조무사, 물리치료사, 방사선사, 약사, 병원 직원, 병원 환자, 외래환자, 입원 환자, 보호자, 병원 관계자, 의원, 한의원, 치과, 약국
Repeated contact	헬스트레이너, 헬스장 강사, PT 트레이너, 요가강사, 필라테스 강사, 운동 트레이너, 운동 코치, 수영강사, 태권도 사범, 관장, 코치

Table S5: Mapping table for relationship categories. Free-text responses recorded under the “etc.” option were harmonized into standardized relationship categories.

Category	Mapping of location categories from free-text responses
Household	기숙사, 학교 기숙사, 기숙사 2인실, 고시원 숙소, 이모집, 조부댁, 외가댁, 할머니댁, 조부모자택, 조부모집, 외갓집, 할아버지댁, 할머니집, 가족집, 작은집, 누나집, 딸집, 거주지, 부모집, 친정집, 부모님 댁, 부모님댁, 아버지댁, 부모님 집, 시댁, 시골집, 처가, 부모닝집, 부모님집, 아들 원룸, 처갓집, 조카네 집, 조카네집 제사, 친정, 처남, 친척집, 엄마집, 아버지집, 부모댁, 확대 가족의 집, 사촌 동생집, 사촌동생집, 이모네 집, 이모네집, 본가(시댁), 본가(친할아버지댁), 본가, 어머니 댁, 가족의 집, 언니 집, 언니네집, 언니집, 동생집, 조카집, 여자친구 집, 당사자 집, 어르신집, 어르신 집, 아이의 집, 돌봄아이의 집, 돌보는 아이의 집, 돌봄 아이의 집, 그 아이 집, 그 친구 집, 지인의집, 친구 집, 친구집, 이웃집, 친구의집, 엄마친구집, 친구네 집, 옆집, 지인 집, 상대방집, 상대방 집, 친구네, 지인집, 다른 사람 집, 당근마켓 거래자집, 집은 상대방집을 의미함, 접촉자 집, 접촉자의 집, 접촉자의집, 2 번째 접촉한 사람의 집, 3 번째 접촉한 사람의 집, 3 번째 접촉자의 집, 2번째 접촉자의 집, 1번째 접촉자의 집, 환자집, 친척, 가족, 이모님
Workplace	사무실, 알바사무실, 사무실 빌딩 휴게실, 사무실 휴게실, 연구소, 사내, 알바 장소, 알바, 알바 현장, 알바하는 장소, 통근버스, 알바 장소 가는 셔틀차, 알바 셔틀차, 사내직원, 업무장소, 회사 엘리베이터
Educational facility	어린이집, 어린이집 기사, 자녀의 어린이집, 유치원, 학원, 피아노 학원, 운전학원, 운전면허학원, 축구교실, 수영장교실, 웰빙노래교실, 노래교실, 합기도장, 신세계백화점 문화센터, 문화센터, 문화센터 교실, 이마트 문화센터, 강의장, 수강생, 교육원, 교육장, 이반장 교육장, 교육장소, 교육기관, 교육시설, 음악실, 학교, 대학교, 강의실, 강당 옆자리, 강당, 통학버스안, 통학버스, 어린이집 버스, 어린이집 차, 유치원차량, 하교차량, 자녀의 활동보조 하교 차량, 자녀의 활동 지원사의 차량안, 봉고차 기사, 학원버스안, 독서실, 반학생, 학원 선생님, 학원강사, 유치원 친구 엄마, 어린이집 차량, 유치원 버스, 교육센터, 하원차량, 과외방, 태권도, 공부방
Religious facility	교회, 성당, 절
Restaurant / Cafe / Bar	식당, 학교 내부의 구내식당, 급식실, 기숙사 식당, 복지관 식당, 한식당, 구내식당, 식당 서빙, 회사식당, 커피점, 장위시장 음식점, 장위음식점, 분식점, 분식집, 까페, 카페, 키즈카페, 애견카페, 헌혈카페, 방탈출 카페, 커피숍, 메가커피, 스터디카페, 스터디까페, 스카, 방탈출, 중국집, 치킨집, 족발집, 진량다향오리, 제주돈돈, 신촌설렁탕 문화점, 식당 직원, 카페직원, 키즈카페친구, 피자집, 커피전문점, 김밥집, 피지집, 맥도날드, 피자 가게, 피자가게, 핫도그 가게, 버거가게, 유흥시설, 분식가게, 보드게임 카페, 포장마차
Hospital	병원, 소아병원, 울산대학병원, 울산대병원, 정형외과 병원, 내과병원, 동물병원, 병원(초음파2시간), 치과병원, 안과병원, 병원진료실, 병원데스크, 병원 진료실, 병원 주사실, 병원물리치료실, 병원 물리치료실, 병원 예약 창구, 병원 검사실, 병원 채혈실, 병원 영상 촬영실, 병원 X-ray촬영실, 병원 원무과, 병원 응급실 접수 창구, 종합병원, 병원약국, 응급실 입원실, 병실, 벼원, 병웜, 병윈, 안과, 정형외과, 소아과, 피부과, 김재은내과(시흥동), 중촌동 박기서내과, 오류동 세계방사선과, 병원(소아과), 외과병원, 외과, 의원, 치과의원, 치괴의원, 치과 병원, 치과 진료실, 치과, 피부과의원, 안과 의원, 안과의원, 호흡기질환의원, 치과 의원, 김안과의원, 내과의원, 내과, 한의원, 약국, 약국병원, 하양약국, 아파트단지 내 버드내약국, 대사동 정문약국, 악국, 한방약국, 병원실장님, 병원 간호사, 간호사, 병원의사, 병원간호사, 병원 심전도 검사직원, 병원 응급실 간호사, 약국직원, 약사, 간호원, 병실간호사실, 요양원, 요양시설, 요양원실급, 요양병원, 보건소, 심혈관 조영술, 심장초음파 검사실, 심혈관 조영술 검사실, 심혈관 초음파실 이동, 병문안, 응급실, 병원 응급실, 대학병원, 병원 접수창구, 정형외과병원, 병원(안과), 동네 병원, 서울삼성병원, 명내과, 성모안과, 김재은내과, 성형외과, 대전중촌동 박기서내과, 산부인과, 정형외과 물리치료실, 신생아실, 입원 환자, 삼성서울병원, 삼성서울병원 회의, 병원 경비실
Outdoor	교외, 야외, 야외(공원), 실외, 밖, 외부, 길거리, 도로, 주차장, 공원, 놀이터, 산, 여행지, 캠핑장, 버스정류장, 시장, 역주변, 동물원, 아파트, 아파트 단지, 아파트 주차장, 길, 골목, 공터, 휴게소, 주유소, 유원지, 롯데월드, 에버랜드, 푸드트럭, 노점상, 과일노점, 모름

Table S6: Mapping table for contact location categories. Free-text responses recorded under the “etc.” option were reclassified into predefined location categories.

	Date	Weekdays	The number of contact (CI 90%)
Round 1	23.12.6.	Tue	6.175 (6.000, 6.350)
	23.12.7.	Wed	5.649 (5.476, 5.823)
	23.12.8.	Thu	5.583 (5.407, 5.760)
	23.12.9.	Fri	4.048 (3.928, 4.168)
	23.12.10.	Sat	3.633 (3.523, 3.743)
	23.12.11.	Mon	5.368 (5.202, 5.534)
	23.12.12.	Thu	5.293 (5.132, 5.454)
Round 2	24.2.7.	Tue	5.449 (4.824, 5.118)
	24.2.8.	Wed	4.971 (4.824, 5.118)
	24.2.9.	Thu	4.745 (4.613, 4.878)
	24.2.10.	Fri	6.012 (5.840, 6.184)
	24.2.11.	Sat	4.229 (4.106, 4.351)
	24.2.12.	Mon	3.478 (3.380, 3.576)
	24.2.13.	Thu	4.623 (4.492, 4.754)

Table S7: Average number with 90% confidence interval of reported contacts per participant, stratified by day of the week.

- **Metropolitan**: Seoul, Incheon, and Gyeonggi-do
- **Chungcheong**: Daejeon, Sejong Special Self-Governing City, Chungcheongbuk-do, and Chungcheongnam-do
- **Gangwon**: Gangwon Special Self-Governing Province
- **Youngnam**: Busan, Daegu, Ulsan, Gyeongsangbuk-do, and Gyeongsangnam-do
- **Honam**: Gwangju, Jeollabuk-do, Jeollanam-do, and Jeju Special Self-Governing Province

These regional groupings were used consistently across quota sampling, descriptive analyses, and population weighting procedures.

Note S4. Population weighting considerations

Post-stratification weights were not applied directly to the released dataset. Instead, we provide the reference population distributions by age group and geographic region (Table S1), which were used during quota sampling.

This design choice reflects the fact that optimal weighting strategies may differ depending on the specific research objective (e.g., age-based transmission modeling versus regional or household-level analyses). By providing the underlying population benchmarks, we enable downstream users to construct customized post-stratification weights appropriate for their own analytical needs, rather than constraining reuse to a single study-defined weighting scheme.
